# Comparative evaluation of a rapid diagnostic test, an antibody ELISA, and a pLDH ELISA in detecting asymptomatic malaria parasitaemia in blood donors in Buea, Cameroon

**DOI:** 10.1186/s40249-017-0314-2

**Published:** 2017-08-01

**Authors:** Tebit Emmanuel Kwenti, Longdoh Anna Njunda, Beltine Tsamul, Shey Dickson Nsagha, Nguedia Jules-Clement Assob, Kukwah Anthony Tufon, Dilonga Henry Meriki, Enow George Orock

**Affiliations:** 1 Department of Medical Laboratory Sciences, Faculty of Health Sciences, University of Buea, P.B. 63, Buea, Southwest Region Cameroon; 2 Department of Public Health and Hygiene, Faculty of Health Sciences, University of Buea, P.B. 63, Buea, Southwest Region Cameroon; 3 Blood Bank, Regional Hospital Buea, P.B. 32, Buea, Southwest Region Cameroon; 4 Programme in Medicine, Faculty of Health Sciences, University of Buea, Buea, Southwest Region Cameroon

**Keywords:** Blood transfusion, Malaria, RDT, pLDH ELISA, Malaria antibody ELISA, Comparative evaluation, Sensitivity, Specificity, Buea, Cameroon

## Abstract

**Background:**

In malaria endemic areas, infected blood donors serve as a source of infection to blood recipients, which may adversely affect their prognosis. This necessitates the proper screening of blood to be used for transfusion in these areas. The purpose of this study was to determine the prevalence of malaria parasitaemia in blood donors in Buea, Cameroon, and to evaluate the performance of a rapid diagnostic test (RDT), a malaria antibody enzyme-linked immunosorbent assay (ELISA), and a *Plasmodium* lactate dehydrogenase (pLDH) ELISA in the detection of asymptomatic malaria parasitaemia in the target population.

**Methods:**

In a prospective study conducted between September 2015 and June 2016, 1 240 potential blood donors were enrolled. The donors were screened for malaria parasites using Giemsa microscopy (GM) and a RDT. A sub-sample of 184 samples, comprising 88 positive and 96 negative samples, were selected for the evaluation of the pLDH ELISA and the antibody ELISA. The chi-square test and correlation analysis were performed as part of the statistical analyses. The statistical significance cut-off was set at *P* < 0.05.

**Results:**

The prevalence of malaria parasitaemia in this study was found to be 8.1% (95% *CI*: 6.6 – 9.7). The prevalence was not observed to be dependent on the age or sex of the participants. The RDT had a sensitivity (88.0%), specificity (99.1%), and negative predictive value (99.0%) higher than the ELISAs. The performance of the pLDH ELISA, which demonstrated the highest positive predictive value (91.6%), was generally comparable to the RDT. The sensitivity was lowest with the antibody ELISA (69.9%), which also demonstrated the highest false positive and false negative rates. The detection threshold for the pLDH (three parasites/μl) was lower compared to the RDT (50 – 60 parasites/μl). Non-significant positive correlations were observed between the parasite density and the pLDH titers and malaria antibody titers.

**Conclusions:**

Overall, the RDT and the pLDH ELISA demonstrated a perfectly correlated agreement with GM, meanwhile the antibody ELISA demonstrated a substantially correlated agreement with GM. The pLDH is therefore recommended for mass screening of blood (to detect malaria parasitaemia) for transfusions in the study area. However, where this is not feasible, an RDT will suffice.

**Electronic supplementary material:**

The online version of this article (doi:10.1186/s40249-017-0314-2) contains supplementary material, which is available to authorized users.

## Multilingual Abstracts

Please see Additional file 1 for translations of the abstract into the five official working languages of the United Nations.

## Background

Malaria is mainly transmitted through the bite of an infected female *Anopheles* mosquito. Transmission can also occur through transfusion of infected blood. In malaria-endemic areas, epidemiological studies have reported a prevalence of malaria among potential blood donors to range between 1% and > 50% [1–3]. The risk of *Plasmodium* transmission through blood transfusion is accounted for by the persistence of malaria parasites in the blood; *P. vivax* may persist in blood for one year, *P. ovale* for three years, and *P. malariae* for even longer periods before causing clinical malaria [4, 5]. Blood donors will remain asymptomatic and are therefore a potential source of infection. Moreover, malaria parasites may survive in the red blood cells at refrigerator temperatures (2 – 4 °C) for days or weeks [6]. It is well established that all five *Plasmodium* species known to cause disease in humans may also be transmitted through blood transfusion [7]. Transmission of malaria through blood transfusion may adversely affect the prognosis of the recipient, especially those with a weakened immune system as in HIV patients. This necessitates the proper screening of blood for transfusion.

The detection of malaria parasites in Giemsa-stained thick and thin blood films by light microscopy is considered the gold standard for malaria diagnosis [8–12] and it is currently the most widely used technique for the diagnosis of malaria in endemic areas [13]. The technique is highly sensitive and specific when in expert hands and can detect as little as five parasites/μl of blood [14]. Some of the technique’s shortcomings are that it is labour intensive, time-consuming, and requires substantial training and expertise [8–12, 14–16], which might not be readily available in endemic areas. A number of methods have been described to improve the sensitivity of malaria microscopy [17], and new diagnostic methods have been developed such as rapid diagnostic tests (RDTs), enzyme-linked immunosorbent assays (ELISAs), and polymerase chain reaction (PCR) assays [14, 16, 18].

However, the newer diagnostic techniques are also not without problems. Some of these assays including ELISAs and PCR are very expensive, and require hi-tech facilities in addition to trained expertise. Yet, these assays do have the added advantage of filling the gap left by the shortage of expert microscopists. In addition, ELISAs have wide applicability needed for mass screening of blood for the detection of malaria parasitaemia within a short period in endemic area. As such, light microscopy in the diagnosis of malaria has no place in blood banking since it is labour intensive and time consuming for the examination of a large number of samples [7, 19]. There is therefore a need for the development of simple, rapid, easy to perform, and reliable methods, as well as the continuous evaluation of currently existing malaria diagnostic assays for their application in blood banking, in order to reduce the transmission of malaria during blood transfusions.

This study’s objectives were to determine the prevalence of malaria among blood donors in Buea, and to compare the performance of a RDT, a *Plasmodium* lactate dehydrogenase (pLDH) antigen ELISA, and a malaria antibody ELISA in the detection of asymptomatic malaria parasitaemia in potential blood donors in Buea.

## Methods

### Study area

Buea (4°10’0”N 9°14’0”E) with an elevation of 870 m (2 854 ft) is located in the eastern slopes of Mount Cameroon. Buea is the capital of the Southwest Region of Cameroon. The population of Buea is estimated at 200 000 [20] and it is considered one of the fastest growing towns in Cameroon today, with a cosmopolitan setting and a constellation of about 67 villages. Buea has two seasons — the dry season (between October and March), and the rainy season (between April and September). Human malaria can be described as mesoendemic in the dry season and hyperendemic in the rainy season, with peaks at the beginning and towards the end of the rainy season [21]. The population of Buea experiences an estimated 3.93 infective bites/person/night [22]. *P. falciparum* accounts for up to 96% of malaria infections in this area [23].

### Study design and setting

This study was a prospective study, in which potential blood donors were recruited at the blood bank of the Regional Hospital of Buea between September 2015 and June 2016. This blood bank serves as a reference blood bank in the Southwest Region. The centre receives donors from Buea and its surrounding villages.

### Sample size estimation

To compare the two ELISAs, the sample size was estimated using the following formula [24]:

$$ n=\frac{{\left[{Z}_{\frac{\alpha}{2}}\sqrt{2\times \overline{P}\left(1-\overline{P}\right)}+{Z}_{\beta}\sqrt{P_1\left(1-{P}_1\right)+{P}_2\left(1-{P}_2\right)}\right]}^2}{{\left({P}_1-{P}_2\right)}^2} $$

where:

*n* = sample size for sensitivity or specificity;

*Z*_α/2_ = normal distribution value based on a 95% confidence limit of 1.96;

*P*_1_ = sensitivity and specificity of NovaLlisa™ Malaria antibody ELISA;

*P*_2_ = sensitivity and specificity of DRG Malaria antigen ELISA; and

*Z*_β_ = 0.84.

From previous studies, the sensitivity (P_1_) and specificity (P_1_) of NovaLisa™ Malaria antibody ELISA were 89% and 91.6% respectively [25], meanwhile the sensitivity (P_2_) and specificity (P2) of DRG Malaria antigen ELISA were 100% and 100% respectively [26].

Using power (1–β) = 80%, α = 0.05, and confidence interval (*CI*) = 95%, the sample sizes for the sensitivity and specificity were 67 positive samples and 89 negative samples, respectively.

To determine the number of donors to be screened, the number of positive samples needed was divided by the prevalence of 6.5% for malaria in blood donors in Yaoundé, Cameroon [27], i.e. 67/0.065, which gave a sample size of at least 1 031 potential blood donors.

### Study population

Potential blood donors in the Regional Hospital of Buea were approached to take part in the study. The inclusion criteria were male or female patients aged between 17 and 55 years. All participants were expected to provide a signed informed consent, which was duly explained to them in English, French, or the local broken English. Excluded from the study were individuals who did not meet the criteria for blood donation (and were therefore deemed unfit).

### Sampling technique

A convenient sampling technique was used in which potential blood donors were consecutively recruited from the blood bank of the Regional Hospital of Buea.

### Laboratory analysis

#### Specimen collection

Capillary and venous blood samples were collected from participants who gave consent. Capillary blood was used to perform the malaria RDT and the preparation of thick and thin blood films for malaria microscopy. Venous blood was collected into EDTA anticoagulated tubes to conduct a complete blood count (CBC) and perform ELISA.

### Conducting a CBC

The CBC was conducted using the Mindray® BC-2800 Auto Hematology Analyzer (Mindray Bio-Medical Electronics Co., Ltd, Shenzhen, China). The white blood cell (WBC) counts were obtained from the CBC results and used for the estimation of the malaria parasite density.

### Malaria diagnosis

#### Diagnosis by Giemsa microscopy (GM)

The prepared blood films were air-dried and stained with 10% Giemsa (1 in 20 dilutions) for 25 – 30 min [28]. Detection of malaria parasites and estimation of the parasite density by light microscopy was performed as described elsewhere [29]. Two expert microscopists read the blood films separately, not knowing each other’s results. In case of any discrepancy with the results obtained by the two microscopists, a third was brought in and the results given in that case were considered as final. The thick films were screened for at least 200 fields using the 100X (oil immersion) objective. If asexual stages of *Plasmodium* were seen, they were counted until 500 WBCs were reached. The slides were only declared negative after counting to 2 500 WBCs. Malaria parasite density was estimated by dividing the parasites counted by 500 and then multiplied by the actual WBC count of the participant (obtained from the CBC result) to give numbers in parasite per μl [29].

#### Diagnosis by RDT

A commercially available RDT kit (*CareStart*™ Malaria HRP2/pLDH [Pf/PAN] Combo, ACCESSBIO, INC., New Jersey, USA) was used to detect malaria parasites, according to the manufacturer’s instructions, using 5 μl of capillary blood. The membrane strips were read and interpreted after 20 min, as described elsewhere [30].

#### Diagnosis by antibody and antigen detection ELISAs

Commercial ELISA kits were used to diagnose malaria in the study population: the NovaLisa™ Malaria antibody ELISA (NovaTec Immundiagnostica, GmbH, Germany), which detects antibodies raised against *Plasmodium* antigens, MSP1 and CSP coated on the microtiter wells; and the DRG Malaria Ag ELISA (DRG International Inc., New Jersey, USA), which detects pLDH, as according to the manufacturer’s instructions (a detailed description can be found in the Additional file 2). The antibody or antigen titres were read using an ELISA plate reader, the BioTek® ELx800^TM^ (BioTek Instruments, Inc., Vermont, USA) at 450 nm against a reference wavelength of 620 nm.

For the antibody ELISA, the cut-off value was calculated by taking the average of the four cut-off absorbance values obtained. In order to convert the result into arbitrary units, the patient’s mean absorbance value was multiplied by 10 and divided by the cut-off value. Samples were considered positive if the result in units was greater than 11 (absorbance value is higher than 10% over the cut-off) and samples were considered negative if the result in units was less than 9 (absorbance value is lower than 10% below the cut-off). An equivocal result was obtained when the result fell between 9 and 11 unit.

For the pLDH ELISA detection, the cut-off value was computed by multiplying the optical density of the three negative control titres by 3. The antigen index (AI) was obtained by dividing the optical density of the samples by the cut-off value. An AI ≤ 0.8 was considered negative, an AI between 1.0 and 0.8 was considered as equivocal, and an AI ≥ 1.0 was considered positive. All samples with equivocal results were tested again for confirmation.

### Data analysis

Data was entered into an Excel spreadsheet and analysed using IBM® SPSS® Statistics version 20 (IBM, USA). Frequency tables were used to present demographic characteristics. ELISA titres and malaria parasite densities were log transformed prior to analysis. Correlation analyses were performed to measure the association between ELISA titres and malaria parasite density. The Pearson’s chi-square test was used to compare group proportions. Using GM as the gold standard, each of the technique’s (RDT, antibody-detecting ELISA, and the antigen-detecting ELISA) sensitivity, specificity, false positive rate (FPR), false negative rate (FNR), positive predictive value (PPV), negative predictive value (NPV), and measure of agreement with GM were deduced.

All equivocal results were considered as positive. The detection limit was calculated from the sample with the lowest parasitaemia with the true positive result. The strength of agreement was categorized as follows: poorly correlated (<0%), slightly correlated (0 – 20%), fairly correlated (21 – 40%), moderately correlated (41 – 60%), substantially correlated (61 – 80%), and perfectly correlated (81 – 100%). Statistical significance was set at *P* < 0.05.

## Results

One thousand, two hundred and forty (1 240) potential blood donors successfully took part in this study. Out of these, 1 132 (91.3%) were males and 108 (8.7%) were females. The participants were aged between 17 and 52 years (mean ± SD = 32 ± 7.81) (see Table 1).

**Table 1:** Distribution of malaria prevalence in the study population according to age and gender

Parameter		*n*	Prevalence of malaria *n* (%)	Chi-square (*χ* ^2^)	*P-*value
Gender	Male	1 132	92 (8.1)	0.069	0.794
	Female	108	8 (7.4)		
Age	<20	22	4 (18.2)	8.833	0.065
	20 – 29	373	29 (7.8)		
	30 – 39	516	40 (7.8)		
	40 – 49	312	23 (7.4)		
	≥50	17	4 (23.5)		

The participants were screened for malaria parasites using GM. Among the participants, 100 were positive for malaria, giving an overall prevalence of 8.1% (95% *CI*: 6.6 – 9.7). No significant association was observed between the prevalence of malaria and sex (*P* = 0.794). Likewise, no significant association was observed between the prevalence of malaria and age (*P* = 0.065) (see Table 1). In this study, 94 (94%) of infected patients had *P. falciparum* malaria; four (4%) had mixed infection with *P. falciparum* and *P. malariae*; and two (2%) had mixed infection with *P. falciparum* and *P. ovale*.

Out of the 100 samples that were positive, as determined by GM, 88 were also found positive by the RDT. Out of the 1 140 samples that were negative, as determined by GM, 1 126 were also found negative by the RDT and 14 were found positive. Out of the 88 positive samples selected for evaluation, 69 were also found positive using the pLDH ELISA and 68 were found to be positive using the malaria antibody ELISA. Among the 96 selected negative samples, 89 were also found to be negative using the pLDH ELISA and 65 were found to be negative using the malaria antibody ELISA (see Table 2).

**Table 2:** Summary of results obtained with the pLDH ELISA, malaria antibody ELISA, and RDT

		GM		
		Positive *n* (%)	Negative *n* (%)	Total *n* (%)
RDT	Positive	88 (86.2)	14 (13.7)	102 (8.2)
	Negative	12 (1.1)	1 126 (99.0)	1 138 (91.8)
	Total	100 (8.1)	1 140 (91.90)	1 240 (100)
pLDH ELISA	Positive	69 (94.5)	4 (5.5)	73 (39.7)
	Negative	12 (19.8)	89 (88.1)	101 (54.9)
	Equivocal	7 (70)	3 (30)	10 (5.4)
	Total	88 (47.8)	96 (52.2)	184 (100)
Malaria antibody ELISA	Positive	68 (73.4)	24 (26.1)	92 (50)
	Negative	16 (19.8)	65 (80.3)	81 (44)
	Equivocal	4 (36.4)	7 (63.6)	11 (6)
	Total	88 (47.8)	96 (52.2)	184 (100)

Among the three diagnostic methods evaluated, the sensitivity was highest with the RDT (88.0%) and lowest with the malaria antibody ELISA (69.9%). A similar trend was observed with the specificity and the NPV. With respect to the PPV, the highest value was observed with the pLDH ELISA (91.6%) and the lowest with the antibody ELISA (81.8%). Among the three methods, the FPR was highest with the antibody ELISA and lowest with the RDT. The FNR was highest with the antibody ELISA and lowest with the pLDH ELISA. Overall, a perfectly correlated agreement with GM was observed with the RDT (97.9%) and the pLDH ELISA (89.7%). Meanwhile, a substantially correlated agreement was observed with the antibody ELISA (74.5%) (see Table 3).

**Table 3:** The performance of the pLDH ELISA, malaria antibody ELISA, and the RDT for the detection of malaria parasites, as compared with the microscopic method (GM)

Parameter	pLDH ELISA % (*CI*)	Antibody ELISA % (*CI*)	RDT % (*CI*)
Sensitivity	86.0 (77.4 – 92.8)	69.9 (60.1 – 78.6)	88.0 (80.0 – 94.0)
Specificity	92.7 (85.6 – 97.0)	80.3 (69.9 – 88.3)	99.1 (98.0 – 99.3)
PPV	91.6 (83.4 – 96.5)	81.8 (72.2 – 89.2)	89.8 (78.0 – 92.3)
NPV	88.1 (80.2 – 93.7)	67.7 (57.4 – 76.9)	99.0 (98.2 – 99.5)
FPR	7.3 (5.8 – 9.1)	19.7 (17.3 – 22.3)	1.2 (0.6 – 2.1)
FNR	2.3 (1.5 – 3.4)	30.1 (27.3 – 33.1)	12 (6.4 – 20.0)
Agreement between tests	89.7 (84.4 – 93.7)	74.5 (67.5 – 80.6)	97.9 (96.9 – 98.6)

Sensitivity = [true positive/(true positive + false negative) × 100]; specificity = [true negative/(true negative + false positive) × 100; PPV = [true positive/(true positive + false positive) × 100]; NPV = [true negative/(true negative + false negative) × 100]; Agreement = [true positive + true negative/N × 100]; FPR = 1 ˗ specificity; FNR = 1 ˗ sensitivity

All four (100%) cases of mixed infection with *P. falciparum* and *P. malariae* were detected by the RDT, pLDH ELISA, and malaria antibody ELISA. The two (100%) cases of mixed infection with *P. falciparum* and *P. ovale* were detected by the RDT and malaria antibody ELISA, meanwhile only one (50%) was detected by pLDH ELISA.

**Fig. 1:**
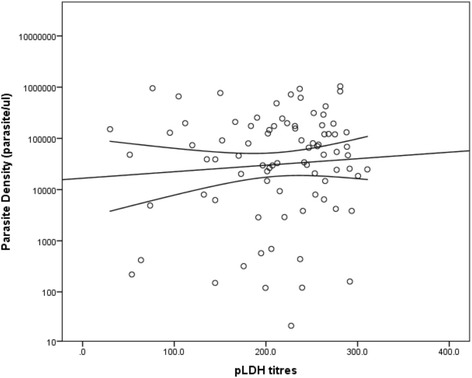
Plot of parasite density against pLDH titres. A weak positive correlation was observed between parasite density and pLDH titers (*r* = 0.04, *P* = 0.720)

**Fig. 2:**
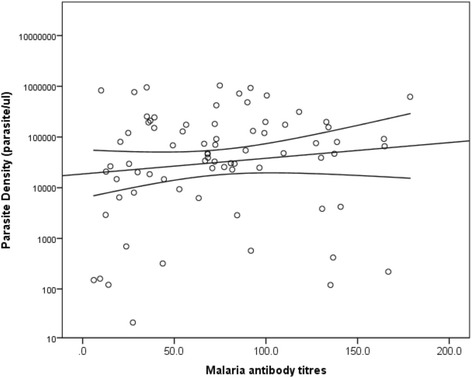
Plot of parasite density against malaria antibody titres. A weak positive correlation was observed between parasite density and antibody titers (*r* = 0.182, *P* = 0.118)

The geometric mean parasite density (GMPD) was 24.96 parasites/μl (95% *CI*: 9.28–67.1). The detection threshold of the RDT was 50 – 60 parasites/μl, meanwhile the detection threshold was approximately three parasites/μl for the pLDH ELISA. Compared to GM, a non-significant positive correlation was observed with the pLDH antigen titres (*r* = 0.04, *P* = 0.720, see Fig. 1) and the malaria antibody titres (*r* = 0.182, *P* = 0.118, see Fig. 2).

## Discussion

A prevalence of 8.1% for malaria was observed in the study population. The prevalence observed in this study was comparable to other studies conducted in Yaoundé (6.5%) and Ghana (10%) [27, 31]. On the contrary, a lower prevalence has been reported among blood donors in Ethiopia (1%) [3] and a higher prevalence among blood donors in Nigeria (29.4%) [32]. The difference in the malaria prevalence reported in these studies and ours could be attributed to the differences in the endemicity of malaria; in the present study area, malaria transmission varies between holoendemic to hyperendemic and affects mostly children [21, 33]. The malaria prevalence observed among blood donors in this study was lower compared to the malaria prevalence (25.6%) reported in the general population of Buea [34]. As mentioned earlier, malaria in the study area is more common in children, however, adults and teenagers were targeted in this study, which could account for the lower prevalence observed.

In the current study, no significant association was observed between the prevalence of malaria and age or gender. The finding of no association between malaria prevalence and age corroborates studies performed elsewhere [32, 35]. The finding of no association between malaria prevalence and gender is in conformity with a study conducted by Oladeinde et al. [35], but contrary to a study conducted by Oche and Aminu [32], in which malaria prevalence was observed to be significantly higher in females.

Out of the three malaria diagnostic assays evaluated, the RDT demonstrated the highest sensitivity, specificity, NPV, and lowest FPR compared to the ELISAs. Furthermore the RDT had the highest agreement with the GM (97.9%). In addition, it is easy to use and has the shortest turnaround time of approximately 25 min. Between the two types of ELISAs, the pLDH ELISA demonstrated optimal performances in terms of the sensitivity, specificity, PPV, and the NPV compared to the malaria antibody ELISA. These findings are in conformity to a study performed in Benin [36]. In addition, the agreement between the ELISAs and GM was observed to be higher with the pLDH ELISA (89.7% vs. 74.5%). The differences in the performance of the ELISAs could be explained by their underlying principle of operation; the antigen ELISA is a sandwich ELISA detecting pLDH antigen, a glycolytic pathway enzyme secreted by different *Plasmodium* species [37]. The pLDH enzyme is known to disappear from the circulation within 24 h of effective treatment [38]. Therefore, the pLDH is considered a specific marker for the presence of viable *Plasmodium* in the blood and is therefore used for screening in malaria-endemic countries. On the other hand, the antibody ELISA detects antibodies raised against the malaria parasites and is therefore dependent on the inherent ability of the individual to raise antibodies against the parasites, which may be absent during the acute phase of the infection and may also be a problem in immunodeficient patients. Antibody detection ELISAs are also more prone to cross-reactivity with antibodies raised against other infectious agents. Furthermore, antibodies produced against malaria parasites may take a longer time to wane. These are some of the factors that may have influenced the performance of the malaria antibody ELISA in this study, which limits its usefulness in the diagnosis of malaria, especially in areas of high transmission. Similar observations have been reported in other studies [5, 36].

In the current study, the majority of donors had infection only with *P. falciparum*, followed by mixed infection with *P. falciparum* and *P. malaria,* and mixed infection with *P. falciparum* and *P. ovale*. The distribution of the *Plasmodium* species were similar to that reported by Kwenti et al. [21]. All the cases of mixed infections with *P. falciparum* and *P. malariae* were found to be positive with both the pLDH ELISA and the malaria antibody ELISA, with the exception of one case of mixed infection with *P. falciparum* and *P. ovale*, which was not found to be positive with the pLDH ELISA. Although the pLDH enzyme is produced by all *Plasmodium* species, there are different species-specific isomers that may not be detected by the pLDH-based ELISAs. However, this finding should be interpreted with caution since just two samples were positive for mixed infection with *P. ovale*. The low detectability of *P. ovale* by pLDH ELISAs has, however, previously been reported [36, 39].

In the current study, the GMPD of malaria was 24.96 parasites/μl. The detection threshold of the pLDH ELISA was three parasites/μl. This is not too dissimilar from the study conducted by Atchade et al. [36], which demonstrated that pLDH ELISA was able to detect parasite density as low as one parasite per ml of blood. On the other hand, the detection threshold of the RDT was 50–60 parasites/μl. This diminishes the value of RDT for use in the screening of blood for blood transfusion as donors are often asymptomatic and may therefore have a very low parasite density. The low sensitivity of the RDT in detecting low malaria parasitaemia has previously been reported by Maltha et al. [30]. This observation demonstrates the ability of the pLDH ELISA to detect parasites at a much lower concentration compared to the RDT and therefore reinforces its application in blood transfusions.

Although the malaria antibody titres and the pLDH titres were observed to increase with increasing parasite density, no significant correlation was observed between the GMPD and pLDH titres (*P* = 0.720) or the antibody titres (*P* = 0.118) in this study. This therefore implies that the ELISAs cannot be used for direct quantification of malaria parasitaemia in the target population.

In this study, ELISA was not performed on all 1 240 samples collected due to the costs involved, which serves as a major limitation. Furthermore, the samples on which ELISA assays were performed were not screened for the presence of *Mycoplasma* species, rubella, Epstein-Barr virus, rheumatoid factor, and other pathologies known to be associated with cross-reactivity of ELISAs.

## Conclusions

In conclusion, a substantial prevalence of malaria was observed among blood donors in the study area, which therefore presents a risk of transfusion-associated malaria. The prevalence of malaria was not observed to be dependent on the age or the gender of the participants. Overall, the RDT performed better than the ELISAs in all aspect except for the PPV. Between the two ELISAs, the pLDH ELISA demonstrated optimal performance in terms of the sensitivity, specificity, PPV, and NPV compared to the malaria antibody ELISA and its agreement was perfectly correlated to GM. In addition to a higher PPV, the pLDH ELISA had a lower detection threshold (three parasites/μl) compared to the RDT. Thus, we recommended using a pLDH ELISA for the mass screening of blood (to detect asymptomatic malaria parasitaemia) for transfusion in the study area. However, where this is not feasible, using a RDT will suffice.

## Additional files

Additional file 1: Multilingual abstracts in the five official working languages of the United Nations. (PDF 550 kb)

Additional file 2: Detail description of the protocol for the pLDG ELISA and malaria antibody ELISA. (DOCX 13 kb)

## Abbreviations

AI: Antigen index
CBC: Complete blood count
*CI*: Confidence interval
ELISA: Enzyme-linked immunosorbent assay
FNR: False negative rate
FPR: False positive rate
GM: Giemsa microscopy
GMPD: Geometric mean parasite density
NPV: Negative predictive value
PCR: Polymerase chain reaction
pLDH: *Plasmodium* lactate dehydrogenase
PPV: Positive predictive value
RDT: Rapid diagnostic test
WBC: White blood cell
