# Supplementary material for: Comparative evaluation of a rapid diagnostic test, an antibody ELISA, and a pLDH ELISA in detecting asymptomatic malaria parasitaemia in blood donors in Buea, Cameroon
Source: Infect Dis Poverty. 2017 Aug 1;6:103. doi: 10.1186/s40249-017-0314-2 (PMC5537946; doi:10.1186/s40249-017-0314-2)
Supplement: Supplementary file 1 — Multilingual abstracts in the five official working languages of the United Nations. (PDF 550 kb) [file 40249_2017_314_MOESM1_ESM.pdf]

Translation of the abstract into the five official working languages of the United Nations

تقييم مقارن لاختبار تشخيصي سريع، واختبار مقايسة المُمْتَزِ المناعي المرتبط بالإنزيم للأجسام المضادة، واختبار مقايسة المُمْتَزِ المناعي المرتبط بالإنزيم للمستضد pLDH في الكشف عن طفيليات الملاريا في الدم بدون أعراض في المتبرعين بالدم في بويا، الكاميرون

تابت ايمانويل كوينتي، لونجدو آنا نجوندا، بيلتين تسامول، شي ديكسون نساجا، نجيديا جول كليمنت أسوب، كوكوا أنتوني توفون، ديلونجا هنري ميريكي، إينوو جورج أوروك

ملخص

خلفية: في المناطق الموبوءة بالملاريا، يكون المتبرعون بالدم المصابين مصدرا للعدوى في المتبرع لهم، الأمر الذي قد يؤثر سلبا على تشخيصهم. وهذا يتطلب فحص الدم جيدا لاستخدامه في نقله للمحتاجين في هذه المناطق. وكان الغرض من هذه الدراسة هو تحديد مدى انتشار طفيليات الملاريا في المتبرعين بالدم في بويا، الكاميرون، وتقييم أداء اختبار التشخيص السريع، واختبار مقايسة المُمْتَزِ المناعي المرتبط بإنزيم الأجسام المضادة للملاريا، واختبار مقايسة المُمْتَزِ المناعي المرتبط بالإنزيم للمستضد pLDH في الكشف عن طفيليات الملاريا في الدم بدون أعراض في السكان المستهدفين.

الطريقة: في دراسة استطلاعية أجريت بين سبتمبر 2015 ويونيو 2016، تم تسجيل 1240 متبرعا محتملا بالدم. تم فحص المتبرعين بحثا عن طفيل الملاريا باستخدام صبغة جميزا المجهري (GM) واختبار التشخيص السريع. وقد تم اختيار عينة فرعية من 184 عينة، تضم 88 عينة إيجابية و96 عينة سلبية، لتقييم اختبار مقايسة المُمْتَزِ المناعي المرتبط بالإنزيم للمستضد pLDH واختبار مقايسة المُمْتَزِ المناعي المرتبط بالإنزيم للأجسام المضادة. تم إجراء اختبار خي مربع وتحليل الارتباط كجزء من التحليلات الإحصائية. تم تعيين الحد المعين للدلالة الإحصائية عند P <0.05.

النتائج: وجد أن معدل انتشار الطفيليات الملاريا في هذه الدراسة بلغ 8.1٪ (95٪ CI: 6.6-9.7). لم يلاحظ اعتماد الانتشار على عمر أو جنس المشاركين. كانت حساسية اختبار التشخيص السريع (88.0٪)، والنوعية (99.1٪)، والقيمة التنبؤية السلبية (99.0٪) أعلى من اختبارات مقايسة المُمْتَزِ المناعي المرتبط بالإنزيم. كان أداء اختبار مقايسة المُمْتَزِ المناعي المرتبط بالإنزيم للمستضد pLDH، والذي أظهر أعلى القيمة التنبؤية الإيجابية (91.6٪)، متوافق عموما مع اختبار التشخيص السريع. وكانت الحساسية أقل في اختبار مقايسة المُمْتَزِ المناعي المرتبط بالإنزيم للأجسام المضادة (69.9٪)، والذي أظهر أيضا أعلى معدلات سلبية كاذبة وإيجابية كاذبة. وكان مستوى الكشف عن المستضد pLDH (ثلاثة طفيليات / ميكرولتر) أقل مقارنة مع اختبار التشخيص السريع (50 - 60 طفيلي / ميكرو لتر). وقد لوحظت علاقة طردية غير ذات دلالة إحصائية بين كثافة الطفيل والعيارات الحجمية للمستضد pLDH والعيارات الحجمية للأجسام المضادة للملاريا.

الاستنتاجات: عموما، أظهر اختبار التشخيص السريع واختبار مقايسة المُمْتَزِ المناعي المرتبط بالإنزيم للمستضد pLDH اتفاقا مترابطا تماما مع صبغة جميزا، وأظهر اختبار مقايسة المُمْتَزِ المناعي المرتبط بالإنزيم للأجسام المضادة في الوقت نفسه اتفاقا مترابطا بشكل كبير مع صبغة جميزا. لذا ينصح بإجراء pLDH للفحص الشامل للدم (للكشف عن الطفيليات الملاريا) للدم المنقول في منطقة الدراسة. ومع ذلك، عندما لا يكون ذلك ممكنا، يمكن الاكتفاء باختبار التشخيص السريع.

Translated from English version into Arabic by Free bird, through

TRANSLATORS WITHOUT BORDERS

快速诊断试剂、抗体 ELISA 和 pLDH ELISA 在喀麦隆布埃亚献血者中检测无症状疟疾中的比较评价

Tebit Emmanuel Kwenti, Longdoh Anna Njunda, Beltine Tsamul, Shey Dickson Nsagha, Nguedia Jules-Clement Assob, Kukwah Anthony Tufon, Dilonga Henry Meriki and Enow George Orock

摘要
引言：在疟疾流行区，受感染的献血者可作为传染源将疾病传播给受血者，这同时还会影响对病人的诊断。因此，非常有必要对该地区用于输血的血液样本进行筛查。本研究的目的是测定喀麦隆布埃亚献血者中疟疾的流行率，并且评估快速诊断试剂、抗体 ELISA 和 pLDH ELISA 在目标人群中检测无症状疟疾的表现。
方法：在 2015 年 9 月至 2016 年 7 月开展的前瞻性研究中，共纳入 1240 名献血者。通过吉姆莎染色镜检和 RDT 进行疟原虫筛查。一亚组为 184 名病例最终被纳入评估 pLDH ELISA 和抗体 ELISA，包括 88 名阳性和 96 名阴性。采用卡方检验和相关分析进行统计分析，临界值为 $P < 0.05$。
结果：本研究发现疟疾流行率为 8.1%（95% *CI*: 6.6 – 9.7）。疟疾流行率与年龄和性别无关。RDT 的敏感性为 88.0%，特异性为 99.1%，阴性预测值为 99.0%，高于 ELISAs。pLDH ELISA 的阳性预测值最高为 91.6%，与 RDT 相当。抗体 ELISA 的敏感性最低为 69.9%，同时它的假阳性率和假阴性率也最高。pLDH ELISA 的临界检测值低于 RDT。原虫密度和 pLDH 滴度和疟疾抗体滴度之间没有明显的相关性。
结论：总体而言，RDT 和 pLDH ELISA 与吉姆莎染色结果高度一致，抗体 ELISA 与吉姆莎染色结果基本一致。因此，在该地区可以将 pLDH 推广作为针对输血的群体筛查项目。然而如果可行性较差，RDT 也是足够满足需求的。

Translated from English version into Chinese by Men-Bao Qian, through
CHINESE CENTER FOR DISEASE CONTROL AND PREVENTION
NATIONAL INSTITUTE OF PARASITIC DISEASES

Evaluation comparative d'un test de diagnostic rapide, d'un anticorps ELISA, et d'un pLDH ELISA dans la détection d'une parasitémie du paludisme asymptomatique chez les donneurs de sang à Buea, au Cameroun

Tebit Emmanuel Kwenti, Longdoh Anna Njunda, Beltine Tsamul, Shey Dickson Nsagha, Nguedia Jules-Clement Assob, Kukwah Anthony Tufon, Dilonga Henry Meriki and Enow George Orock

RESUME
Contexte: Dans les régions endémiques du paludisme, les donneurs de sang contaminé sont une source de contamination pour les personnes qui reçoivent ce sang, ce qui peut affecter défavorablement leur pronostic. Cela nécessite qu'un dépistage sanguin approprié soit effectué pour les transfusions dans ces régions. L'objectif de cette étude était de déterminer la prédominance d'une parasitémie du paludisme chez les donneurs de sang à Buea, au Cameroun, et d'évaluer la performance d'un test de diagnostic rapide (TDR), d'un essai de détection d'un anticorps d'immuno-absorption enzymatique du paludisme (ELISA), et de lactate déshydrogénase de *Plasmodium* (pLDH*)* ELISA dans la détection d'une parasitémie asymptomatique du paludisme dans la population cible.
Méthodes: Dans une étude estimative effectuée entre Septembre 2015 et Juin 2016, 1240 donneurs de sang potentiels ont été inscrits. On a analysé le sang des donneurs pour un dépistage de parasites du paludisme en utilisant la microscopie au Giemsa (MG) et un TDR. Un sous-échantillon de 184

échantillons, comprenant 88 échantillons positifs et 96 échantillons négatifs, a été sélectionné pour l'évaluation du pLDH ELISA et de l'anticorps ELISA. Le test du chi carré (χ2) et l'analyse de corrélation ont été réalisés comme partie des analyses statistiques. Le seuil de signification statistique a été fixé à $P < 0,05$.

Résultats: Dans cette étude, on a trouvé que la prédominance d'une parasitémie du paludisme était de 8,1% (95% *CI*: 6,6 – 9,7). La prédominance ne s'est pas avérée être dépendante de l'âge ou du sexe des participants. Le TDR avait une sensibilité (88,0%), une spécificité (99.1%), et une valeur prédictive négative (99.0%) plus élevées que celles de l'ELISA. La performance du pLDH ELISA, qui a démontré la valeur prédictive positive la plus élevée (91,6%), a été globalement comparable au TDR. La sensibilité a été moins élevée avec l'anticorps ELISA (69,9%), qui a également démontré les taux les plus élevés de faux positifs et faux négatifs. Le seuil de détection du pLDH (trois parasites/µl) a été moins élevé comparé au TDR (50 – 60 parasites/µl). Des corrélations positives non-significatives ont été observées entre la densité parasitaire et les titres du pLDH et les titres de l'anticorps du paludisme.

Conclusions: Dans l'ensemble, le TDR et le pLDH ELISA ont démontré un accord parfaitement corrélé avec la MG, et en même temps l'anticorps ELISA a démontré un accord substantiellement corrélé avec la MG. Le pLDH est par conséquent recommandé pour le dépistage de masse du sang (afin de détecter une parasitémie du paludisme) pour les transfusions dans la région de l'étude. Cependant, lorsque cela n'est pas faisable, un TDR suffira.

Translated from English version into French by Ode Laforge, through

TRANSLATORS
WITHOUT BORDERS

Сравнительная оценка быстрых диагностических тестов, антитела ELISA, и pLDH ELISA при выявлении бессимптомной паразитемией малярии в крови доноров в городе Буэа, Камерун

Tebit Emmanuel Kwenti, Longdoh Anna Njunda, Beltine Tsamul, Shey Dickson Nsagha, Nguedia Jules-Clement Assob, Kukwah Anthony Tufon, Dilonga Henry Meriki and Enow George Orock

Реферат

Фон: В эндемичных по малярии районах, зараженных доноров крови служат источником инфекции для реципиентов крови, что может негативно сказаться на их прогнозе. Это требует соответствующего анализов крови, используемых для переливания в этих областях. Целью данного исследования было определить распространенность малярии паразитемией в крови доноров в городе Буэа, Камерун, и для оценки выполнения диагностических экспресс-тестов (RDT), иммуноферментный анализ (ELISA) по малярии и антителу, и Плазмодий лактатдегидрогеназы (pLDH) ELISA на выявление бессимптомной паразитемией малярии в целевой группе населения.

Методы: В проспективном исследовании, проведенном в период с сентября 2015 г. по июнь 2016 г., были зачислены 1 240 потенциальных доноров крови. Доноры были обследованы на

возбудителей малярии с помощью микроскопии Giemsa (GM) и RDT. Выборки из 184 образцов, в которые включают 88 положительных и 96 отрицательных образцов, были выбраны для оценки на pLDH ELISA и антитела ELISA. Chi-square тест и корреляционный анализ проводились в рамках статистического анализа. Статистическая отсечения значимости была установлена на уровне $p < 0,05$.

Результаты: Распространенность паразитемией малярии в этом исследовании было обнаружено, составляет 8,1% (95% *CI*: 6.6 – 9.7). Распространенность не зависит от возраста или пола участников. RDT имели чувствительность (88,0%), специфичность (99,1%) и отрицательная прогностическая ценность (99,0%) выше, чем ELISAs. Выступление для pLDH ELISA, которое продемонстрировало высокую положительную прогностическую ценность (91,6%), которая в целом сопоставима с RDT (50 – 60 parasites/µl). Не-значительные положительные корреляции наблюдались между плотностью паразита и pLDH титрами и титрами малярийного антитела.

Заключение: В целом, RDT и pLDH ELISA показал, что прекрасно коррелируется соглашение с GM, тем временем антитела ELISA продемонстрировали существенное коррелированное соглашение с GM. Поэтому pLDH рекомендуется для массового скрининга крови (для выявления паразитемией малярии) для переливания в исследуемой области. Однако там, где неосуществимо, тогда RDT справится.

Translated from English version into Russian by Hao-Qi Zhang

Evaluación comparativa de un test de diagnóstico rápido, un ELISA de anticuerpos y un ELISA pLDH en la detección de parasitemia de malaria asintomática en donantes de sangre en Buea, Camerún

Tebit Emmanuel Kwenti, Longdoh Anna Njunda, Beltine Tsamul, Shey Dickson Nsagha, Nguedia Jules-Clement Assob, Kukwah Anthony Tufon, Dilonga Henry Meriki y Enow George Orock

RESUMEN

Antecedentes: En las áreas endémicas de malaria, los donantes de sangre infectados son una fuente de infección para los receptores de sangre, lo que puede afectar negativamente a su pronóstico. Esto requiere que en estas áreas la sangre que vaya a ser utilizada para transfusión sea analizada adecuadamente. El propósito de este estudio fue determinar la prevalencia de la parasitemia de la malaria en donantes de sangre en Buea, Camerún, y evaluar un test de diagnóstico rápido (TDR), un ensayo por inmunoabsorción ligado a enzimas (ELISA) y un ELISA *Plasmodium* lactato deshidrogenasa (pLDH) en la detección de la parasitemia asintomática de la malaria en la población diana.

Métodos: En un estudio prospectivo realizado entre septiembre de 2015 y junio de 2016, se inscribieron 1240 donantes de sangre potenciales. Los donantes fueron examinados para detectar parásitos de malaria utilizando la tinción de Giemsa (GM) y un TDR. Se seleccionó una submuestra de 184 muestras, que comprendían 88 muestras positivas y 96 negativas, para evaluar el ELISA pLDH y el ELISA de anticuerpos. Se realizaron la prueba de ji-cuadrado y los análisis de correlación como parte de los análisis estadísticos. El valor estadístico de corte se estableció en $P < 0,05$.

Resultados: La prevalencia de la parasitemia de la malaria en este estudio fue de 8,1% (IC del 95%: 6,6 - 9,7). No se observó que la prevalencia dependiera de la edad o del sexo de los participantes. El TDR tuvo una sensibilidad (88,0%), especificidad (99,1%) y valor predictivo negativo (99,0%) mayor que el ELISA. El ELISA pLDH, que demostró tener el mayor valor predictivo positivo (91,6%), fue comparable al TDR. La sensibilidad fue la más baja con el anticuerpo ELISA (69,9%), que también dio los falsos positivos y falsos negativos más altos. El umbral de detección para el pLDH (tres parásitos/µl) fue menor en comparación con el TDR (50-60 parásitos/µl). Se observaron correlaciones positivas no significativas entre la densidad del parásito y los títulos de pLDH y los títulos de anticuerpos contra el paludismo.

Conclusiones: En general, el TDR y el ELISA pLDH mostraron una perfecta concordancia y correlación con la GM, mientras que el anticuerpo ELISA mostró una sustancial concordancia y correlación con la GM. Por lo tanto, se recomienda el pLDH para el cribado masivo de sangre (y detectar la parasitemia de la malaria) de las transfusiones en el área de estudio. Sin embargo, cuando esto no sea posible, un TDR será suficiente.

Translated from English version into Spanish by Amparo Muñoz, through

TRANSLATORS
WITHOUT BORDERS
