# Supplementary material for: Comparative evaluation of a rapid diagnostic test, an antibody ELISA, and a pLDH ELISA in detecting asymptomatic malaria parasitaemia in blood donors in Buea, Cameroon
Source: Infect Dis Poverty. 2017 Aug 1;6:103. doi: 10.1186/s40249-017-0314-2 (PMC5537946; doi:10.1186/s40249-017-0314-2)
Supplement: Supplementary file 2 — Detail description of the protocol for the pLDG ELISA and malaria antibody ELISA. (DOCX 13 kb) [file 40249_2017_314_MOESM2_ESM.docx]

**Description of the protocol for the antibody ELISA**

Briefly, all samples were first of all diluted 1 in 101 in tubes using ready-to-use sample diluents. The tubes were then thoroughly mixed and 100μl of controls (negative, cut-off, positive) and diluted samples were dispensed into microtiter strip wells. The negative control was dispensed into well B, cut-off control into two wells, C and D, and the positive control into well E. All diluted samples and controls were dispensed into wells in duplicate. The plate was then covered in aluminum foil supplied in the kit and incubated for one hour at 37˚C. Afterwards, the content of the wells were aspirated and discarded before being washed three times with 300μl of washing solution (diluted 1 in 20 times), using an automated plate washer, BioTek^®^ ELx50^TM^ (BioTek Instruments, Inc., USA). After washing, 100μl malaria conjugate was added into all wells excluding the blank well (A) and covered with the foil, and incubated for 30 minutes at room temperature. The plate was then removed from the foil after incubation, contents aspirated and discarded, and washed 3 times with washing solution before adding 100μl of TMB substrate into all wells and incubated at room temperature in the dark for 15 minutes. After incubation, the stop solution (containing 0·2M sulphuric acid solution) was added into all wells in the same order and at the same rate as for the TMB substrate solution. The absorbance was measured within 30 minutes at 450nm, with 620nm as the reference wavelength using an ELISA plate reader, BioTek^®^ ELx800^TM^ (BioTek Instruments, Inc., USA). The cut-off value was then calculated by taking the average of the 4 cut-off absorbance obtained. In order to convert the result into units (where Units = NTU), the patient’s mean absorbance value was multiplied by 10 and divided by the cutoff value. Samples were considered positive if the result in units was greater than 11 NTU (absorbance value is higher than 10% over the cut-off) and samples were considered negative if the result in units is less than 9 NTU (absorbance value is lower than 10% below the cut-off). An equivocal result was obtained when the result in units fall between 9 and 11 NTU.

**Description of the protocol for the pLDH ELISA**

Briefly, 100ul of lysing buffer were dispensed into all the wells of a 96 wells microtiter plate. 50ul of reconstituted controls (positive and negative), and homogenized fresh blood samples collected in EDTA anticoagulated tubes were added in to the wells; the negative control was added in triplicate wells meanwhile the positive control was added into a single well. The plate was then covered with self-adhesive plate cover foil supplied and incubated for 60mins at 37°C under continuous gentle shaking condition. Afterwards the content of the wells were emptied by aspiration and washed five times using 350ul of the washing buffer (diluted 1 in 20), with soak time interval of 1mins in-between washes using an automated plate washer, BioTek^®^ ELx50^TM^ (BioTek Instruments, Inc., USA). 100ul of the conjugate solution 1 was added into each well, covered, and incubated at 37°C for 30mins without shaking. The content of the wells was then emptied and washed 5 times as described above. Afterwards, 100ul of conjugate solution 2 was added into each well, covered, and incubated at 37°C for 15mins. After the incubation, the plate was then washed 5 times as described above before adding 100ul of chromogen solution into the wells, covered and incubated at 37°C for 15mins in the dark. Subsequently, 50ul of stopping solution was added to all the wells and the absorbance was read within 15mins using an ELISA plate reader, BioTek^®^ ELx800^TM^ (BioTek Instruments, Inc., USA) at 450nm against a reference wavelength of 630nm. The cut-off value was computed by multiplying the optical density (OD) of the 3 negative control titres by 3. The antigen index (AI) was obtained by dividing the OD of the samples by the cut-off value. AI ≤0.8 was considered negative, AI between 1.0 and 0.8 was considered as equivocal and AI ≥ 1.0 was considered as positive. All samples with equivocal results were repeated once more for confirmation.
